# Neonicotinoid-induced signature dysbiosis identified via metagenomic sequencing of the honey bee gut microbiome

**DOI:** 10.1038/s41598-025-30907-4

**Published:** 2025-12-13

**Authors:** Lan Tran, Thomas B. Deckers, Jonathan Ho, Lance Lansing, Morgan Cunningham, Nuria Morfin, Mateus Pepinelli, Alvaro De la Mora, Ida M. Conflitti, Amanda Gregoris, Linzhi Wu, Daphne Trepanier-Leroux, Laura Muntz, Tara Newman, Shefali Vishwakarma, Miriam Bixby, Hosna Jabbari, Ernesto Guzman-Novoa, Shelley E. Hoover, Robert W. Currie, Stephen F. Pernal, Pierre Giovenazzo, Leonard J. Foster, Amro Zayed, Rodrigo Ortega Polo, M. Marta Guarna

**Affiliations:** 1 Beaverlodge Research Farm, Agriculture and Agri-Food Canada, Beaverlodge, AB Canada; 2 Lethbridge Research and Development Centre, Agriculture and Agri-Food Canada, Lethbridge, AB Canada; 3 Department of Computer Science, University of Victoria, Victoria, BC Canada; 4 Department of Biomedical Engineering, University of Alberta, Edmonton, AB Canada; 5 School of Environmental Sciences, University of Guelph, Guelph, ON Canada; 6 Department of Entomology, University of Manitoba, Winnipeg, MB Canada; 7 Department of Biology, York University, Toronto, ON Canada; 8 School of Natural Sciences, Laurentian University, Sudbury, ON Canada; 9 Department of Biochemistry & Molecular Biology, University of British Columbia, Vancouver, BC Canada; 10 Department of Biological Sciences, University of Lethbridge, Lethbridge, AB Canada; 11 Département de Biologie, Faculté des Sciences et de Génie, Université Laval, Quebec, QC Canada; 12 Present Address: Project Apis m, Salt Lake City, UT 84126 USA

**Keywords:** Honey bees, Microbiota/microbiome, Dysbiosis, Metagenomic sequencing, Neonicotinoids, Metagenomics, Microbiome

## Abstract

The Western honey bee (*Apis mellifera*) plays an essential role in agriculture around the world. In Canada, honey bees contribute up to $7 billion in economic value annually by pollinating crops and producing honey. However, since 2006–2007 North American beekeepers have lost more than a quarter of their colonies each winter. In recent years, the losses have been up to 50% in some regions. The causes of losses are complex, including the interacting effects of nutrition, pathogens, and pesticides. Although the bee gut microbiome plays a crucial role in colony health and disease, studies on the effects of agricultural pesticides on the bee microbial community are sparse. We report the use of shotgun metagenomic sequencing to investigate bee gut microbiota changes, or dysbiosis, in response to two neonicotinoid insecticides, clothianidin and thiamethoxam. Common dysbiosis signatures included an increase in *Bifidobacterium* spp. after chronic sublethal exposure and an increase in *Apibacter adventoris* after short-term acute exposure. Other dysbiosis signatures were unique to each compound, such as an increase in *Snodgrassella alvi* for clothianidin and a decrease in *Lactobacillus* spp. for thiamethoxam. These findings enhance our understanding of how the honey bee gut microbiome responds to stressors and highlight identifiable microbial profile signatures which underscores the potential utility of gut microbiome profiling as a bee health diagnostic tool. Access to timely and accurate bee health diagnosis will inform regulatory actions to decrease and mitigate exposure to stressors and will facilitate managing and improving bee health.

**Supplementary Information:**

The online version contains supplementary material available at 10.1038/s41598-025-30907-4.

## Introduction

The Western honey bee (*Apis mellifera* L.) is an essential pollinator of agricultural crops globally. In Canada, honey bees contribute up to $7 billion per year to the economy through their pollination services and honey production^1^. However, since 2006–2007, the beekeeping industry has suffered losses of over a quarter of their colonies each year. In 2021–2022, Canada lost a record-breaking 45.5% of wintered honey bee colonies^2^, while a recent survey from the United States reported a colony loss of 55.1%^3^. Despite a greater understanding of the various environmental and health stressors that affect bees as a whole^4,5^, studies on the potential impact of stressors on the bee gut microbial community are sparse and are mainly based on 16S ribosomal RNA (rRNA) amplicon sequencing. Metagenomic sequencing is an alternative approach that allows for investigations of microbiomes with increased taxonomic resolution to the level of species and to strain.

The honey bee gut microbiota plays a crucial role in host immunity, learning, memory, and nutrition^6–8^, and can also be an indicator of environmental and bee health^9,10^. Compared with the microbial communities found in livestock and soil^11^, the honey bee gut microbiota is highly conserved and is relatively simple in its composition. Five taxa form the core members of the bee gut microbiota including *Bombilactobacillus* spp. and *Lactobacillus* spp. (formerly members of Firm 4 and Firm 5, respectively^12^), *Gilliamella apicola*, *Snodgrassella alvi*, and *Bifidobacterium* spp^8,13–15^. Other bacterial species may also be present but at lower abundances and include *Frischella perrara* and *Bartonella apis*^15^.

Worldwide, over four million tonnes of agrochemicals (e.g. fungicides, herbicides, insecticides) are used for crop protection each year with over 80% of this usage occurring in the Americas and Asia^16^. However, our dependence on these chemicals is not without potential ecological consequences as their persistence in the environment has been implicated in honey bee colony failure, particularly when combined with other stressors such as nutritional deficiency and disease^4,17,18^. Neonicotinoids, the most commonly used class of insecticides^19^, have been found to affect aspects of bee behaviour and health^18,20–22^, including disrupting homeostasis of the bee gut microbiota^23–25^.

Field studies have shown that neonicotinoid exposure can exceed chronic international levels of concern and potentially result in deteriorated colony health and economic consequences for beekeepers^26^, emphasizing a need to better understand how neonicotinoids affect bees and the bee gut microbiome. A seminal study on glyphosate exposure and its effect on the bee gut microbiota was found to induce dysbiosis in some bacterial strains^27^, which suggests other agrochemicals including neonicotinoids may also disrupt the bee gut microbiota. Clothianidin (CLO) and thiamethoxam (THI) are two neonicotinoids approved for use in Canada^28^ and have been detected in honey bee colonies in agricultural regions such as near corn in Ontario^20^, and in the Fraser Valley in British Columbia^26^. Some field and laboratory studies on the effect of clothianidin or thiamethoxam exposure on the bee gut microbiota have reported shifts in the core taxa including *Lactobacillus* spp^23,29,30^. Previous studies have applied quantitative PCR (qPCR) and 16S rRNA amplicon sequencing to analyze the honey bee gut microbiota’s response to insecticides. In this study, we used shotgun metagenomic sequencing to determine gut microbiota profile changes in response to clothianidin and thiamethoxam after long-term chronic sublethal exposure in colonies and short-term acute sublethal and lethal exposure in laboratory cages. By comparing the microbiota profiles of exposed and control honey bees, we aim to identify specific microbial changes to broaden our understanding of the effects of neonicotinoids on the honey bee gut microbiota, and to potentially use these profile changes as diagnostic signatures of neonicotinoid exposure.

## Results

### Microbiota composition and diversity in experimental bees

**Fig. 1:**
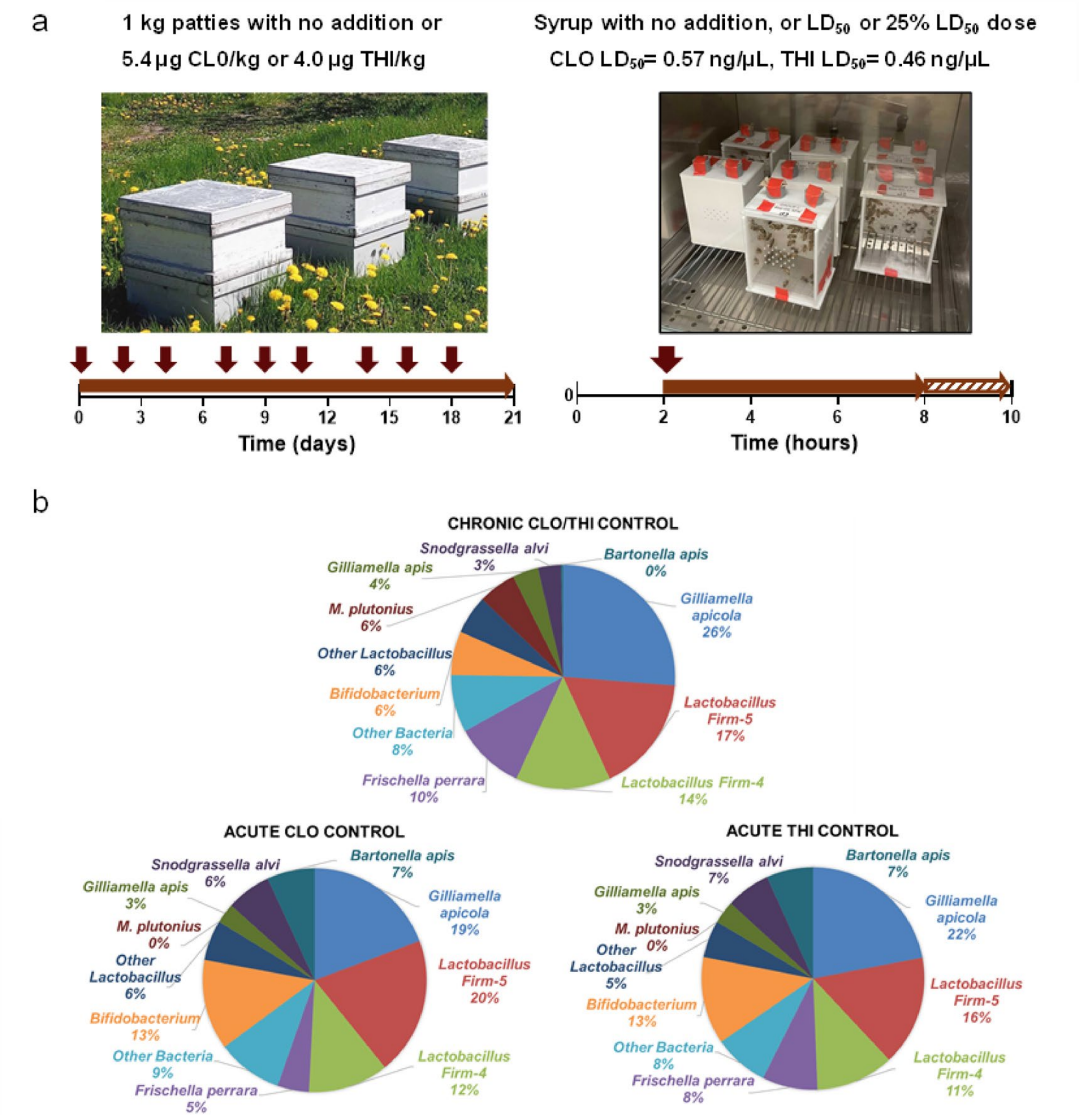
Experimental design and microbial composition of untreated control honey bees. (**a**) Experimental design. For chronic sublethal field exposure experiments in colonies (left), fifteen colonies received pollen patties with or without clothianidin or thiamethoxam (five colonies per treatment). Patties were replaced every 2–3 days (denoted by the arrows) for 21 days. For acute lethal and sublethal experiments in laboratory cages (right), after a 2-hour starvation period, bees were fed a 50% sucrose solution spiked with a lethal dose (LD_50_) or a sublethal dose (equal to 25% of LD_50_) of clothianidin or thiamethoxam for 6 h, followed by 2 h with an untreated 50% sucrose solution. For additional details, refer to the Methods. (**b**) Gut bacteria composition of untreated control bees, as average relative abundances, from experiments testing chronic sublethal clothianidin (CLO) or thiamethoxam (THI) in colonies (top), and acute CLO and THI in cages (bottom).

To examine gut microbiota changes in honey bees exposed to neonicotinoids, bees were exposed to chronic sublethal conditions in colonies, and to acute sublethal and lethal conditions in laboratory cages (Fig. 1a). No differences in survival between controls and treatment groups were observed, as expected, since the experiments were designed to investigate sublethal effects. All colonies survived in the field experiment, and no significant differences in mortality were observed between the control and treatment groups in the cage experiments (clothianidin: H(2) = 5.069, *p* = 0.069; thiamethoxam: H(2) = 5.083, *p* = 0.065; α = 0.05). Shotgun metagenomic sequencing showed the gut microbiota composition of all untreated control bees to be represented by the expected core bacteria: *Bifidobacterium* spp., *Gilliamella* spp., members of *Lactobacillus* Firm 4 and Firm 5 (now known as *Bombilactobacillus* spp. and *Lactobacillus* spp.^12^), and *Snodgrassella alvi* (Fig. 1b). Taxonomic classifications were assigned using the BeeRoLaMa database^31^. *Bartonella apis* and *Frischella perrara*, both of which occur less frequently and are less abundant than the core members^15^, were also detected in the controls. *Bartonella apis* comprised a much lower proportion of the gut microbiota in the chronic colony control than in the acute laboratory cage controls.

**Fig. 2:**
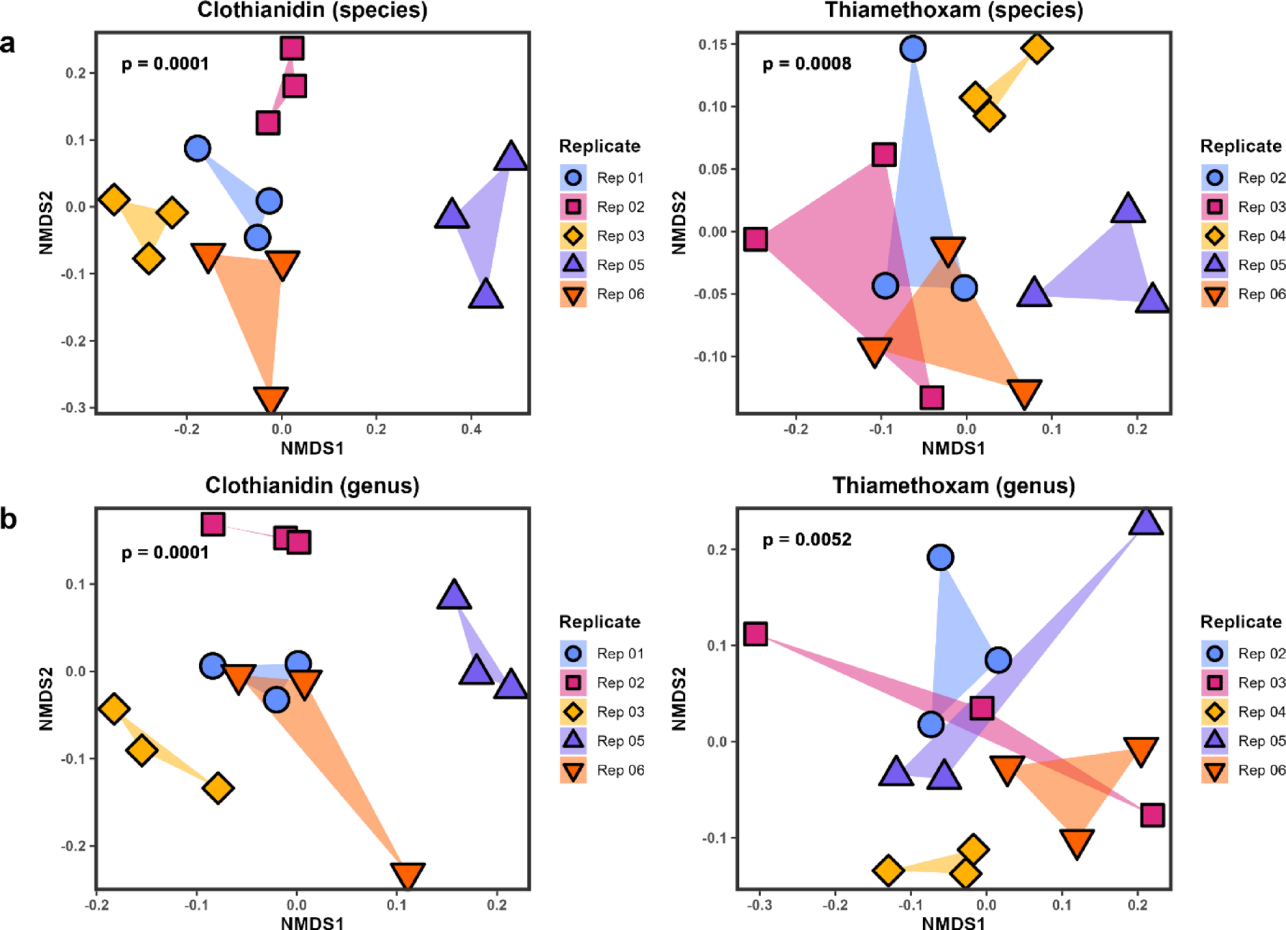
Beta diversity across five honey bee replicates from different source colonies. NMDS plots based on Bray-Curtis dissimilarity for bee gut microbial communities at the (**a**) species level and (**b**) genus level. Bees were exposed to acute sublethal (equal to 25% of LD_50_) and acute lethal (LD_50_) clothianidin or thiamethoxam for 8 h in laboratory cages. Each replicate is denoted by colour and shape, and the three points for each replicate represent control, sublethal, and lethal exposure conditions. Dissimilarity was calculated using proportioned raw reads. The *p*-value for each plot shows the significance of separation grouped by replicate, calculated using ANOSIM with 9,999 permutations. A *p*-value of less than 0.05 was considered statistically significant.

The microbial composition of all experimental bee gut samples was also compared. A beta diversity analysis using non-metric multidimensional scaling (NMDS) based on Bray-Curtis dissimilarity showed significant dissimilarity between laboratory cage replicates at the species and genus levels for the acute sublethal and lethal clothianidin and thiamethoxam experiments (Fig. 2). However, this global analysis did not show significant dissimilarity between treatment conditions for chronic and acute clothianidin or thiamethoxam exposure (Supplementary Figs. S1, S2). These results guided the differential abundance analysis for the gut microbiota detected from neonicotinoid exposure using analysis of compositions of microbiomes with bias correction (ANCOM-BC)^32^. ANCOM-BC was performed considering the source colony for each replicate, i.e. including replicate as a covariate in the analysis.

### Dysbiosis due to neonicotinoid exposure

**Fig. 3:**
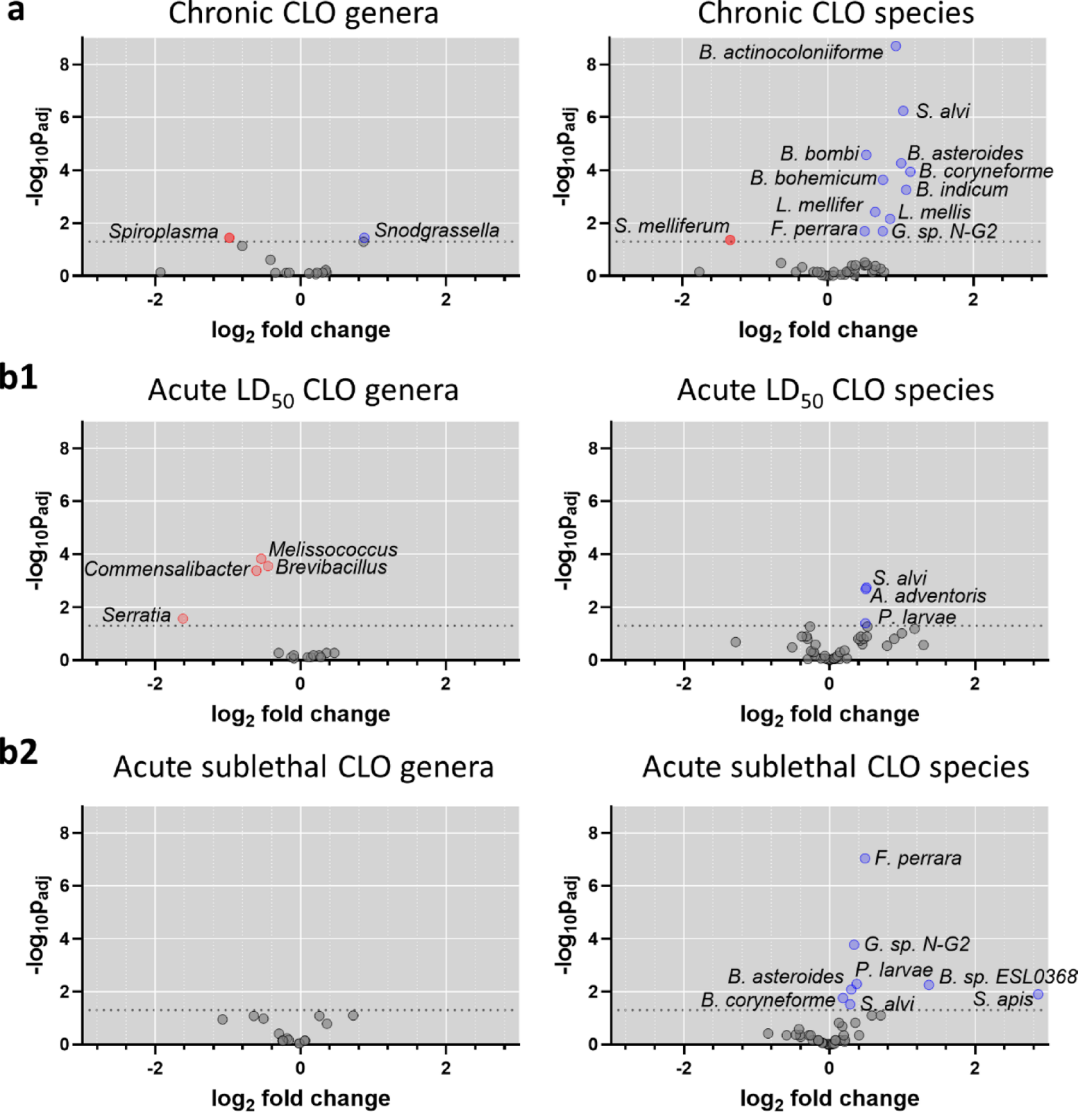
Differential abundance of bacterial species in the gut microbiota of honey bees exposed to clothianidin (CLO). Bees were exposed (**a**) chronically in colonies or acutely in cages at (**b1**) a lethal dose equal to LD_50_, or (**b2**) a sublethal dose equal to 25% of LD_50_. Significance and fold change were determined by ANCOM-BC. For each plot the horizontal dotted line corresponds to an adjusted *p*-value equal to 0.05. Dots above the horizontal dotted line coloured blue represent a significant increase in abundance whereas dots coloured red represent a significant decrease in abundance, compared to the control. For a list of adjusted *p*-values see Supplementary Table ST1. Genera abbreviations: *A: Apibacter*; *B: Bifidobacterium* and *Bombella* for *B.* sp. ESL0368; *F: Frischella*; *G*: *Gilliamella*; *L*: *Lactobacillus*; *P: Paenibacillus*; *S: Snodgrasella* for *S. alvi*, and *Spiroplasma* for *S. apis* and *S. melliferum.*

**Fig. 4:**
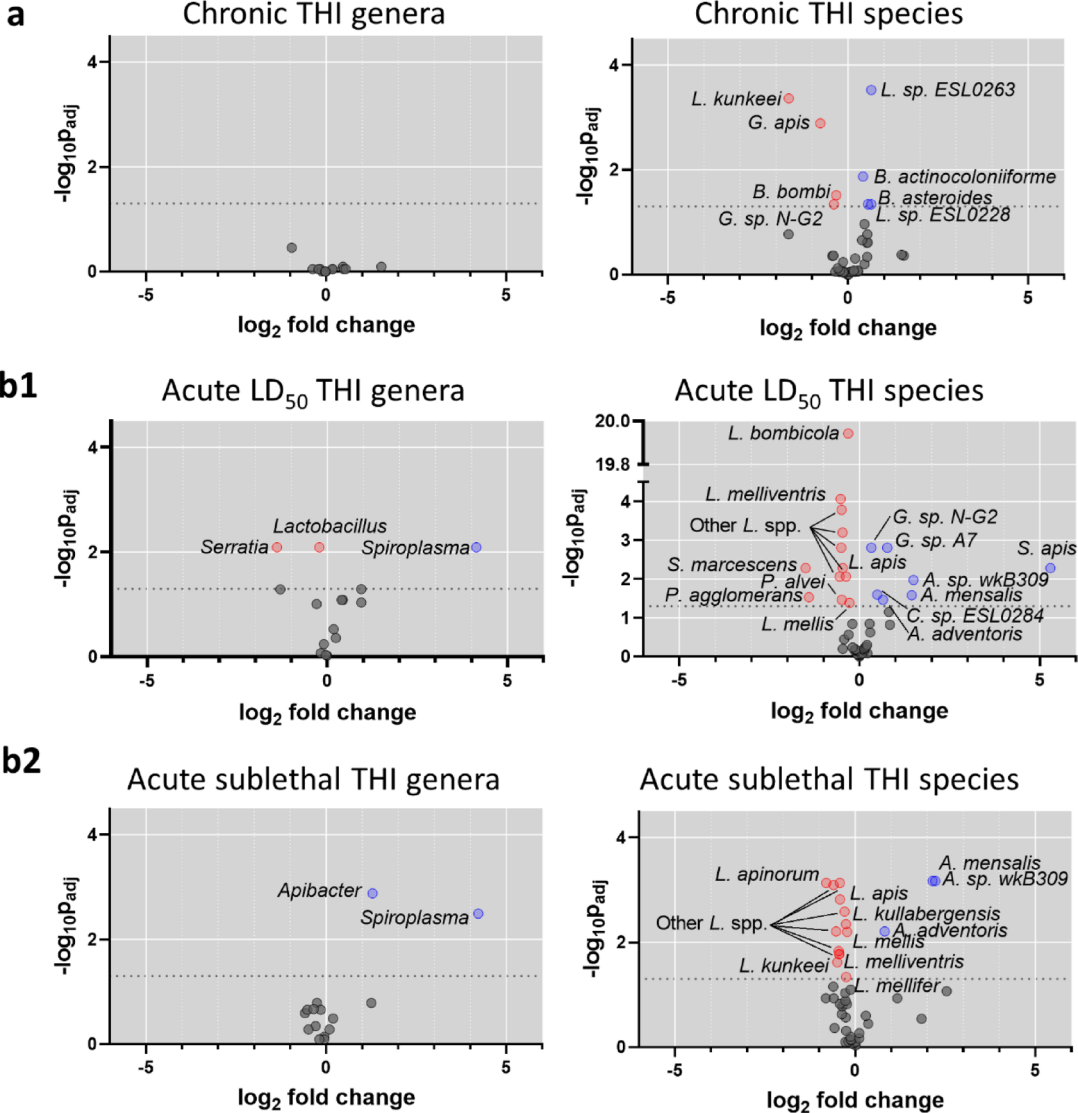
Differential abundance of bacterial species in the gut microbiota of honey bees exposed to thiamethoxam (THI). Bees were exposed (**a**) chronically in colonies or acutely in cages at (**b1**) a lethal dose equal to LD_50_, or (**b2**) a sublethal dose equal to 25% of LD. Significance and fold change were determined by ANCOM-BC. For each plot the horizontal dotted line corresponds to an adjusted *p-*value equal to 0.05. Dots above the horizontal dotted line coloured blue represent a significant increase in abundance whereas dots coloured red represent a significant decrease in abundance, compared to the control. For a list of adjusted *p*-values see Supplementary Table ST1. Genera abbreviations: *A: Apibacter; B: Bifidobacterium*; *C: Commensalibacter*; *G: Gilliamella*; *L: Lactobacillus*; *P: Paenibacillus* for *P. alvei*, and *Pantoea* for *P. agglomerans*; *S: Serratia* for *S. marcescens*, and *Spiroplasma* for *S. apis*.

**Fig. 5:**
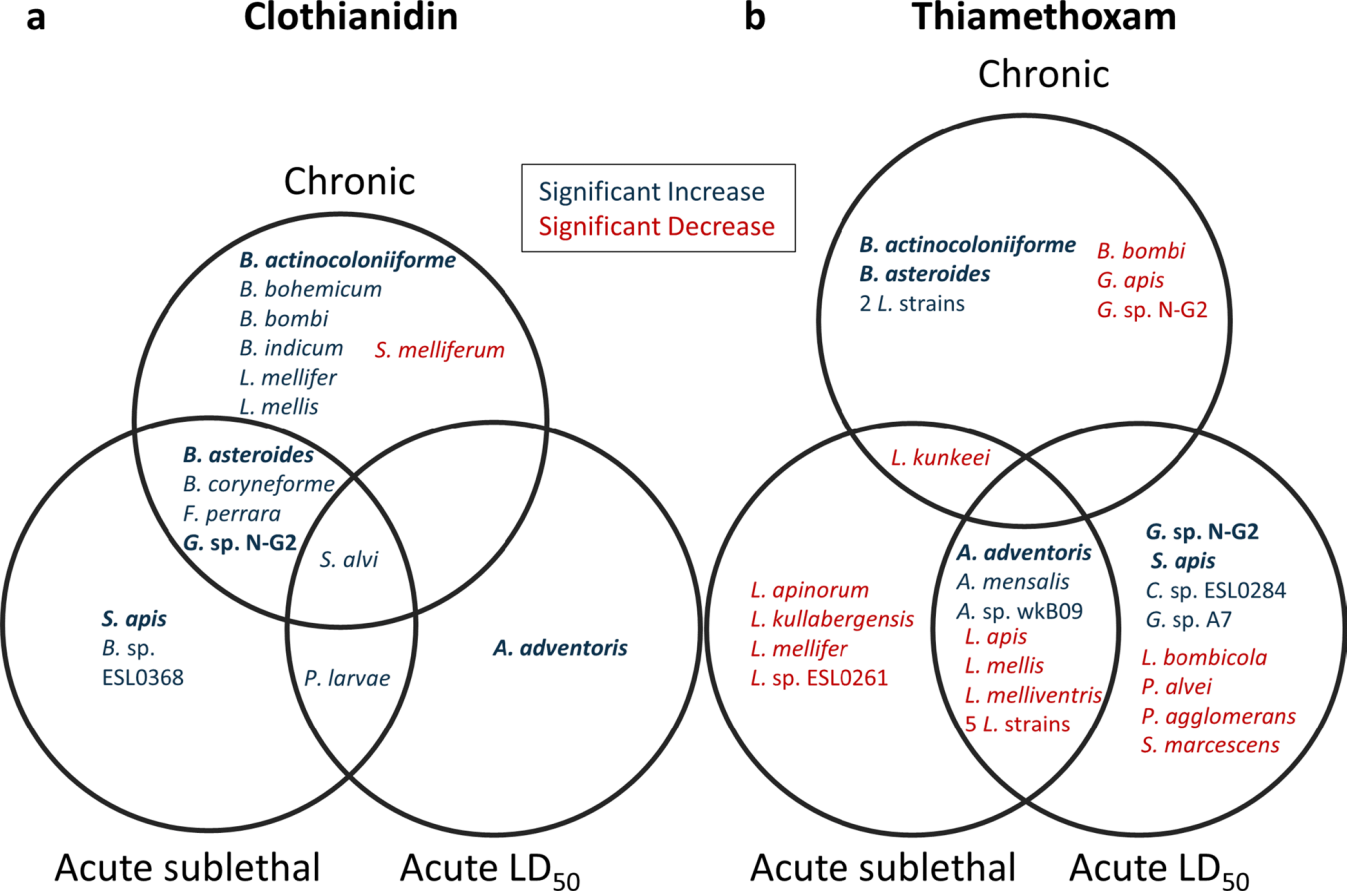
Common and unique significantly differentially abundant microbial species as determined by ANCOM-BC. Differentially abundant species after exposure to (**a**) clothianidin or (**b**) thiamethoxam after chronic sublethal exposure in colonies, or acute exposure at a sublethal (25% of LD_50_) or lethal (LD_50_) dose in cages. Names in bold indicate species that were differentially abundant for both neonicotinoids and the colours indicate whether species abundance significantly increased (blue) or decreased (red). Genera abbreviations: *A: Apibacter*; *B: Bifidobacterium* and *Bombella* for *B.* sp. ESL0368; *C: Commensalibacter*; *F: Frischella*; *G: Gilliamella*; *L: Lactobacillus*; *P: Paenibacillus* for *P. alvei* and *P. larvae*, and *Pantoea* for *P. agglomerans*; *S: Serratia* for *S. marcescens*, *Snodgrassella* for *S. alvi*, and* Spiroplasma* for *S. apis* and *S. melliferum*.

The experimental bees were subjected to three dietary clothianidin or thiamethoxam treatment conditions: chronic sublethal, acute sublethal (at a dose equal to 25% of LD_50_), and acute lethal (LD_50_). Exposure to clothianidin or thiamethoxam resulted in distinct gut microbiota dysbiosis (Figs. 3 and 4, and 5), which was investigated through sequencing followed by taxonomic classification and ANCOM-BC. The common and unique changes in bacterial taxa abundance for the treatment conditions are described below.

### Microbiota signatures identified from chronic and acute clothianidin exposure

Chronic sublethal and acute clothianidin treatments resulted in dysbiosis with overlapping and distinct microbial changes (Fig. 3). A common signature observed for chronic and acute sublethal clothianidin exposures included increases in *Bifidobacterium asteroides*, *Bifidobacterium coryneforme*, *Frischella perrara*, and *Gilliamella* sp. N-G2, while the increase in *Snodgrassella alvi* was a common signature observed for all clothianidin treatments and doses (Fig. 3, Supplementary Table ST1).

Chronic clothianidin exposure resulted in additional shifts with significant increases in *Bifidobacterium* species *B. actinocoloniiforme*, *B. bohemicum*, *B. bombi*, and *B. indicum*, as well as *Lactobacillus* species *L*. *mellifer* and *L. mellis* (Fig. 3). Notably, a decrease in *Spiroplasma melliferum*, a pathogenic bacterium of honey bees, was also observed. Some of the shifts observed at the species level correlated with shifts observed at the genus level, in particular the decrease in the genus *Spiroplasma* and the increase in the genus *Snodgrassella*. Taxa that significantly increased at only an acute dose included *Apibacter adventoris* after acute lethal LD_50_ exposure, and *Bombella* sp. ESL0368 and *Spiroplasma apis* after acute sublethal exposure (Fig. 3, Supplementary Table ST1).

### Microbiota signatures identified from chronic and acute thiamethoxam exposure

Thiamethoxam treatment under the same experimental conditions also induced dysbiosis of the bee gut microbiota. Microbial signatures common to chronic and acute sublethal thiamethoxam exposure included a significant decrease in *Lactobacillus kunkeei*. Acute sublethal and lethal doses resulted in a common increase in *Apibacter* species including *A. mensalis* and *A. adventoris*, the genus *Spiroplasma*, as well as decreases in *L. apis*, *L. mellis*, and *L. melliventris* (Fig. 4, Supplementary Table ST1).

Chronic thiamethoxam exposure resulted in shifts of additional species such as an increase in *B. actinocoloniiforme and B. asteroides*, and a decrease in *B. bombi* and *Gilliamella* sp. N-G2. Acute LD_50_ exposure also resulted in specific changes including a decrease in *L. bombicola*, *Paenibacillus alvei*, *Pantoea agglomerans*, and *Serratia marcescens*, and an increase in *Gilliamella* spp. and *Spiroplasma apis*. After an acute sublethal dose, we observed the genera *Lactobacillus* and *Serratia* decreased in abundance, as well as decreases in additional *Lactobacillus* species including *L. apinorum*, *L. kullabergensis*, and *L. mellifer* (Fig. 4, Supplementary Table ST1). Figure 5 shows common and unique differentially abundant microbial species for all exposure groups.

## Discussion

In our search for dysbiosis signatures diagnostic of neonicotinoid exposure in honey bees, we used shotgun metagenomic sequencing due to its greater taxonomic resolution compared to targeted 16S rRNA sequencing and to avoid primer biases^33^. We found significant microbial changes at both the genus and species levels after exposure to clothianidin and thiamethoxam at chronic sublethal, and acute sublethal and lethal doses. Microbial shifts common to both neonicotinoids included an increase in *Bifidobacterium* spp. after chronic sublethal exposure, and an increase in *Apibacter adventoris* after acute exposure. Other microbial signatures were unique to each compound, which included an increase in *Snodgrassella alvi* after clothianidin exposure, and a decrease in *Lactobacillus* spp. after thiamethoxam exposure.

*Bifidobacterium asteroides* significantly increased in bees exposed to clothianidin and thiamethoxam, but the responses differed depending on the dose. *Bifidobacterium indicum* and *B. coryneforme*, which are closely related species^33,34^, shifted in abundance only when exposed to clothianidin and under chronic and acute sublethal doses. *Lactobacillus* spp. also had contrasting responses to clothianidin and thiamethoxam. Bees exposed to chronic clothianidin significantly increased in *Lactobacillus mellifer* and *L*. *mellis*, whereas other *Lactobacillus* spp. significantly decreased when exposed to acute sublethal and lethal doses of thiamethoxam. Several *Bifidobacterium* species contain glycoside hydrolase gene families involved in carbohydrate degradation^35^ and *Lactobacillus* spp. have been reported to assist in digestion and metabolism of flavonoid glycosides, nucleosides and citrate, and produce beneficial products including organic acids, aromatic compounds and ω-hydroxy acids^36,37^. These two genera have also been linked with bee behavior^38^. The differences in *Bifidobacterium* spp. and *Lactobacillus* spp. in response to clothianidin and thiamethoxam highlights the complexity of the microbiome response when bees are exposed to these stressors. Since *Bifidobacterium* spp., *Lactobacillus* spp., and other members of the bee microbiota have functional cross-interactions^8^, the observed dysbiosis may have additional effects on host health and behaviour by affecting physiological processes or pathogen susceptibility. Further analysis of the metagenomic data reported here will be valuable to assess changes in functional gene abundance and related metabolic pathways, and provide deeper insights into how microbial dysbiosis might affect bee physiology.

Our observed shifts in microbial composition are consistent with previous findings. For example, Rouzé et al. (2019)^23^ found decreases in *Lactobacillus* spp. after sublethal thiamethoxam exposure in caged bees through qPCR quantification of the 16S rRNA gene. Shifts in *Lactobacillus* spp. have also been detected upon exposure to other neonicotinoid compounds, such as during a five-week field experiment in which colonies fed syrup containing imidacloprid or thiacloprid had decreased abundance^24^. Our investigation identified a shift in *Apibacter adventoris*^39^, a low abundance symbiont of the honey bee gut^15^ which increased in response to short-term acute exposure to clothianidin and thiamethoxam. Through amplicon sequencing Jones et al. (2018)^29^ detected an increase in *A. adventoris* in bees whose colonies were located near oilseed rape fields that contained clothianidin and thiamethoxam residues. While further testing is warranted, a shift in *Apibacter adventoris* may be a microbial signature diagnostic of clothianidin or thiamethoxam exposure in honey bees.

Our study also showed dissimilarities with previous reports examining the effect of neonicotinoids on the bee gut microbiota. Non-detectable effects on the gut bacteria were reported when the neonicotinoid imidacloprid was tested by Raymann et al. (2018)^40^, and when thiacloprid was tested by Cuesta-Mate et al. (2021)^25^. Studies by Rouze et al. (2019)^23^ and Alberoni et al. (2021)^24^ reported a decrease in *Bifidobacterium* spp. upon neonicotinoid exposure while our study showed an increase. It is noteworthy that our observed increase in *Bifidobacterium* spp. was measured against an already relatively high baseline level in control bees; *Bifidobacterium* spp. are typically low in abundance compared to the other core members^15^.

The dissimilarities between our results and those from previous studies and the differences observed between our chronic and acute experiments may be explained in a similar manner. As previously noted by Hotchkiss et al. (2022)^41^, pesticide concentration, exposure duration, and concurrent stressors can all affect microbial responses to pesticides. Additionally, dysbiosis outcomes may vary across bee populations due to differences in genetic contributions to neonicotinoid tolerance in honey bees^42^ and by the microbial variation within and between colonies as reported by Rothman et al. (2018)^43^. Because of the latter, we accounted for colony source as a covariate in our analysis. An interesting question is whether microbiota members are capable of metabolizing different classes of neonicotinoids, potentially protecting the bee host from intoxication. However, experiments testing the ability of the microbiota members to degrade imidacloprid found no substantial activity for any of the core gut species^40^. The potential ability of the bee gut microbiome or environmental microbial taxa to metabolize neonicotinoids, as described by Pang et al. (2020)^44^, may help explain differences observed between our colony and cage experiments, as well as discrepancies with earlier studies. These observations underscore the need for further research to validate our findings and to better define the sampling conditions and timing necessary for microbiome profiling to have diagnostic value.

Microbiome shifts may also alter the bee gut pathogenic profile, which may consequently affect bee physiology and health. We found an increase in *Paenibacillus larvae* under acute sublethal and lethal exposure to clothianidin, as well as an increase in *Spiroplasma apis*, and a decrease in *Paenibacillus alvei* and *Serratia marcescens* under acute lethal thiamethoxam exposure. Insecticide exposure has been linked to increased susceptibility to opportunistic pathogens such as *Serratia* in bees exposed to imidacloprid^40^ or to sulfoxaflor^45^. Future studies monitoring the temporal dynamics of symbiotic and pathogenic microbial species in response to stressor exposure will increase our understanding of the functional relevance of the observed shifts. These studies will also inform on whether any of these shifts are compensated, and the overall short- and long-term functional consequences of microbiome profile changes. In conclusion, chronic and acute sublethal and lethal exposure to clothianidin and thiamethoxam resulted in both common and unique responses of the microbiota. Our findings provide additional insight on the direct and indirect effects of neonicotinoids on honey bees and their microbiota. This study also highlights the potential utility of shotgun metagenomic sequencing for identifying microbiota profile signatures of diagnostic value, which could aid in assessing failing colonies and determining the causes of bee health decline.

## Methods

### Chronic sublethal exposure of bees to insecticides

#### Preparation of pollen patties

Pollen patties containing field-realistic concentrations of clothianidin or thiamethoxam were prepared using a method adapted from Tsvetkov et al. (2017)^20^. For neonicotinoid stock solutions, clothianidin (Sigma-Aldrich) or thiamethoxam (Sigma-Aldrich) was dissolved in 1.0 mL of acetone and prepared to a final concentration of 5.35 µg/mL for clothianidin, and 4.0 µg/mL for thiamethoxam. Pollen patties were mixed to contain 56% FeedBee pollen substitute (Dancing Bee Equipment), 33% sugar syrup, and 11% water. For neonicotinoid-containing pollen patties, 1.0 mL of neonicotinoid stock solution was added per 1 kg of pollen patty for a final concentration of 5.35 µg/kg clothianidin and 4.0 µg/kg thiamethoxam. The patties were prepared no more than one day before being deployed to the colonies.

#### Setup, treatment, and sampling of bees in colonies

In July 2020, three colonies for each of each of six replicates were established at the Kortright Centre for Conservation (near Toronto, Ontario, Canada) and at the University of Guelph Honey Bee Research Centre (Guelph, Ontario, Canada). At the beginning of the experiment colonies were randomly assigned to treatments at each site. All colonies had a young, mated, laying queen and were free of visible disease. Each colony had three brood frames, three frames with honey, and four foundation frames. Each colony was also equipped with an activated wooden pollen trap (ApiHex, Model SPT3). Five out of the six colonies were studied for each of the control, clothianidin, and thiamethoxam exposure treatment. A spare colony was in place in the event of queen failure, or another unexpected event. For each replicate, exposed and untreated control colonies were randomly arranged at the same site and faced the same direction. For the experiment, 200 g of pollen patty was provided to each colony and replaced every two to three days for 21 days (Fig. 1A). After 21 days, adult bees were sampled from open brood frames. Bee samples were stored on dry ice in the field and during shipping, and at − 80 °C until processed.

### Acute sublethal and lethal exposure of bees to insecticides

#### Preparation of solutions

Spiked sucrose solutions containing clothianidin or thiamethoxam were prepared using methods adapted from Randhawa et al. (2009)^46^ and Tsvetkov et al. (2019)^20^. For clothianidin, an LD_50_ dose was prepared to a final concentration of 0.57 ng/µL, and a sublethal dose to a final concentration of 0.1425 ng/µL (25% of LD_50_). For thiamethoxam, an LD_50_ dose was prepared to a final concentration of 0.46 ng/µL, and a sublethal dose to a final concentration of 0.115 ng/µL (25% of LD_50_). Both solutions were prepared in 50% sucrose solution and protected from light.

#### Setup, treatment, and sampling of bees in laboratory cages

Three source colonies were located at York University (Toronto, Ontario, Canada) and at the University of Guelph Honey Bee Research Centre (Guelph, Ontario, Canada). Nurse-aged bees were collected from frames containing uncapped brood, from colonies that had a young, mated, laying queen and were free of visible disease. Six replicates were set up for each neonicotinoid experiment and each replicate was sourced from a single colony, i.e. for each replicate, the same source colony was used for both control and treatment groups. Custom plastic cages (Supplementary Fig. S3) were sanitized before use and maintained under complete darkness in an incubator held at 32 ± 2 °C and 50–70% relative humidity. For each acute exposure condition, replicates 1–3 consisted of one cage (two treatments and a control) and replicates 4–6 consisted of two cages, where bees from both cages were later combined for sampling. Each cage housed approximately 60 bees. Bees were starved for two hours, then provided with 4 mL of spiked sucrose solution containing the treatment (Supplementary Table ST2). After six hours of direct exposure, bees were fed a 50% sucrose solution in excess for an additional two hours. At this time, survival was recorded, dead bees were removed from the cage, and the cage was placed on dry ice. A Kruskal-Wallis test was used to compare the proportion of dead worker bees between control and treatment groups. Bee samples were collected and stored at − 80 °C until processed. The replicate with the lowest overall consumption was excluded from analysis.

### Whole gut isolation and shotgun metagenomic sequencing

Gut isolation, sequencing, and taxonomic profiling were performed as previously described^47^. Whole guts were isolated from ten individual bees by excising the head and pulling the stinger; the honey crop and stinger were removed. For each replicate, ten guts were pooled and homogenized using 1 mm glass beads (MilliporeSigma), 2 mL of 1X phosphate buffer saline (PBS), and a 2010 Geno/Grinder homogenizer (SPEX SamplePrep) at 1350 rpm for 30 s. The homogenization step was repeated two to three times and the homogenates were cooled on ice for 30 s between repetitions. All homogenized samples were prepared to a final volume of 3.33 mL in 1X PBS and sent to the Centre d’expertise et des services Génome Québec/Génome Québec Centre of Expertise and Services (Montréal, Quebec, Canada) for genomic DNA extraction, library preparation, and shotgun metagenomic sequencing. Each sequenced sample yielded at least 40 million raw reads. In brief, extracted genomic DNA was quantified using the Quant-iT PicoGreen dsDNA Assay Kit (Life Technologies) and libraries were generated using the NEBNext Ultra II DNA Library Prep Kit for Illumina (New England Biolabs) as per the manufacturer’s recommendations. Size selection of the libraries containing the desired insert size was performed using SparQ beads (Qiagen). Libraries were quantified using the Kapa Illumina GA with Revised Primers-SYBR Fast Universal Kit (Kapa Biosystems). The average fragment size was determined using a LabChip GX Touch Nucleic Acid Analyzer (PerkinElmer). Sequencing was performed on a NovaSeq 6000 (Illumina) and paired-end reads of 151 base pairs were generated.

### Taxonomic profiling and beta diversity analysis

Raw sequences were trimmed of adapters and subjected to quality control using fastp (version 0.22.0)^48^ and aligned to the bacteriophage phiX174 genome (GenBank accession NC_001422.1) using Bowtie 2 (version 2.4.4)^49^. The remaining sequences were aligned to bacterial genomes from the curated BeeRoLaMa database (version 1.0)^31^ for taxonomic classification using Kraken 2 (version 2.1.2)^50^. Taxonomic classifications were according to the accepted names at the time of our analysis. For updated *Lactobacillus* Firm 4 and Firm 5 taxonomic classifications, refer to^12^. Kraken 2 classification reports were processed using the R package Pavian (version 1.0)^51^ to aggregate counts into a single matrix for each experiment. Prior to further analyses, the classification matrices were filtered to contain only bacterial taxa. NMDS coordinates were calculated using the function metaMDS and ANOSIM tests were performed using the function anosim with 9999 permutations. Both functions were from the R package vegan (version 2.5-7)^52^ and were run with the option *distance = “bray”* to use Bray-Curtis dissimilarity as the distance measurement.

### Differential abundance

Differential abundance analysis was performed using the R package ANCOM-BC (version 1.2.2)^32^ with the function ancombc and the option *formula = “treatment + replicate”*. The analysis was repeated to compare each treatment to its corresponding control group. Raw taxon counts were used as the input since the tool implements its own normalization method for sampling fraction correction. Option *p_adj_method = “BH”* was used for multiple testing via the Benjamini-Hochberg method. Taxa with an adjusted *p*-value of < 0.05 were considered to have significant differential abundance.

## Supplementary Information

Below is the link to the electronic supplementary material.

Supplementary Material 1

Supplementary Material 2

## Data Availability

The sequence data from this study are available as Sequence Read Archives (SRAs) under the NCBI BioProject PRJNA1006316, which includes forty-five BioSamples (Supplementary Table S3).
