# Supplementary material for: Neonicotinoid-induced signature dysbiosis identified via metagenomic sequencing of the honey bee gut microbiome
Source: Sci Rep. 2025 Dec 13;16:1211. doi: 10.1038/s41598-025-30907-4 (PMC12789572; doi:10.1038/s41598-025-30907-4)
Supplement: Supplementary file 2 — Supplementary Material 2 [file 41598_2025_30907_MOESM2_ESM.pdf]

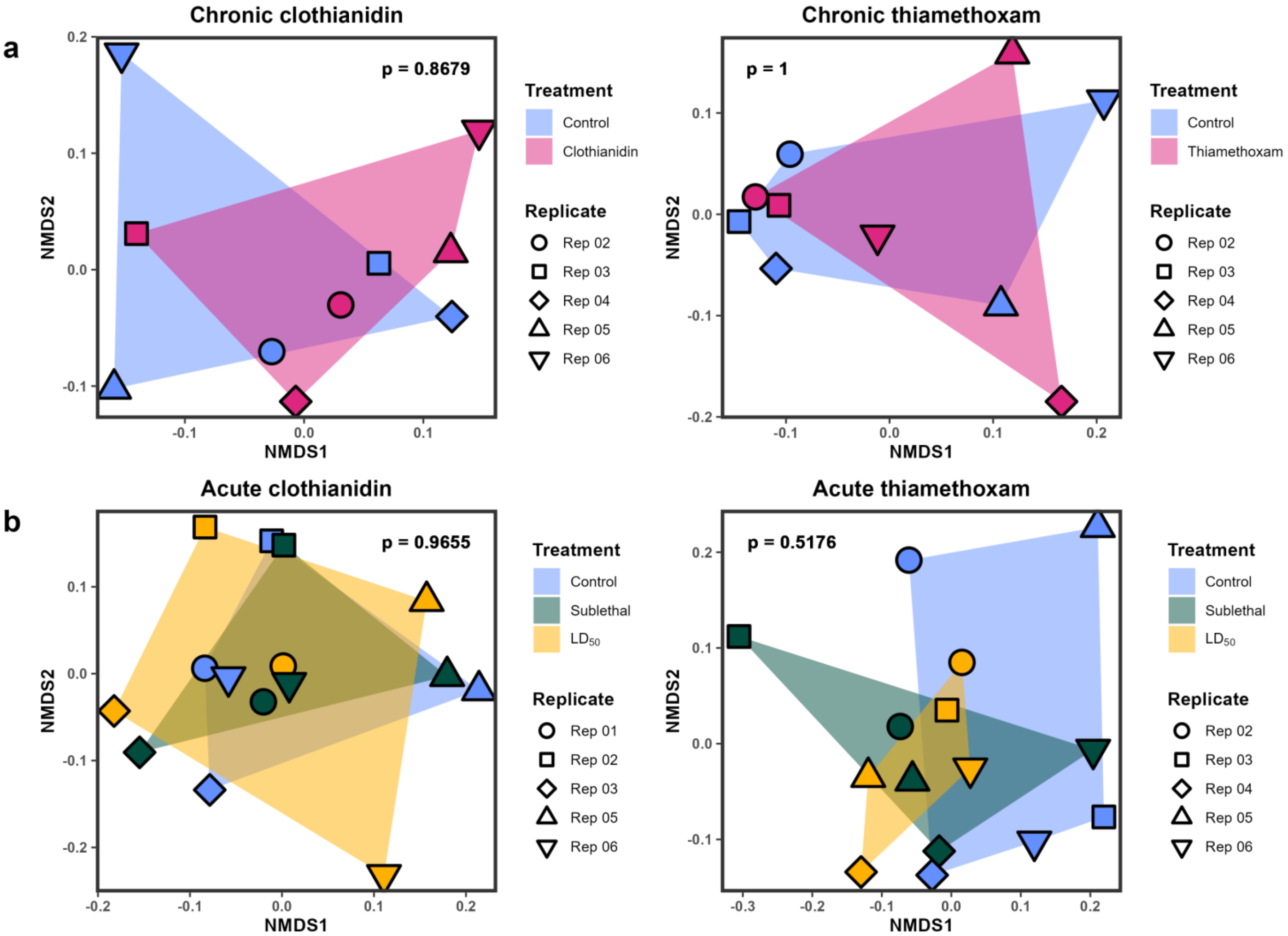

Supplementary Figure S1. NMDS plots based on Bray-Curtis dissimilarity to visualize genus-level beta diversity of honey bees exposed to (a) chronic clothianidin or thiamethoxam in colonies, or (b) acute clothianidin or thiamethoxam in laboratory cages. Each plot is coloured based on treatment: control (blue), chronic clothianidin/thiamethoxam (red), acute sublethal (red), and acute lethal LD_{50} (yellow). The shapes indicate individual replicates. Dissimilarity was calculated using proportioned raw reads and significance was calculated using ANOSIM with 9,999 permutations. The indicated *p*-values for each plot show the significance of separation grouped by treatment. A *p*-value of less than 0.05 was considered statistically significant.

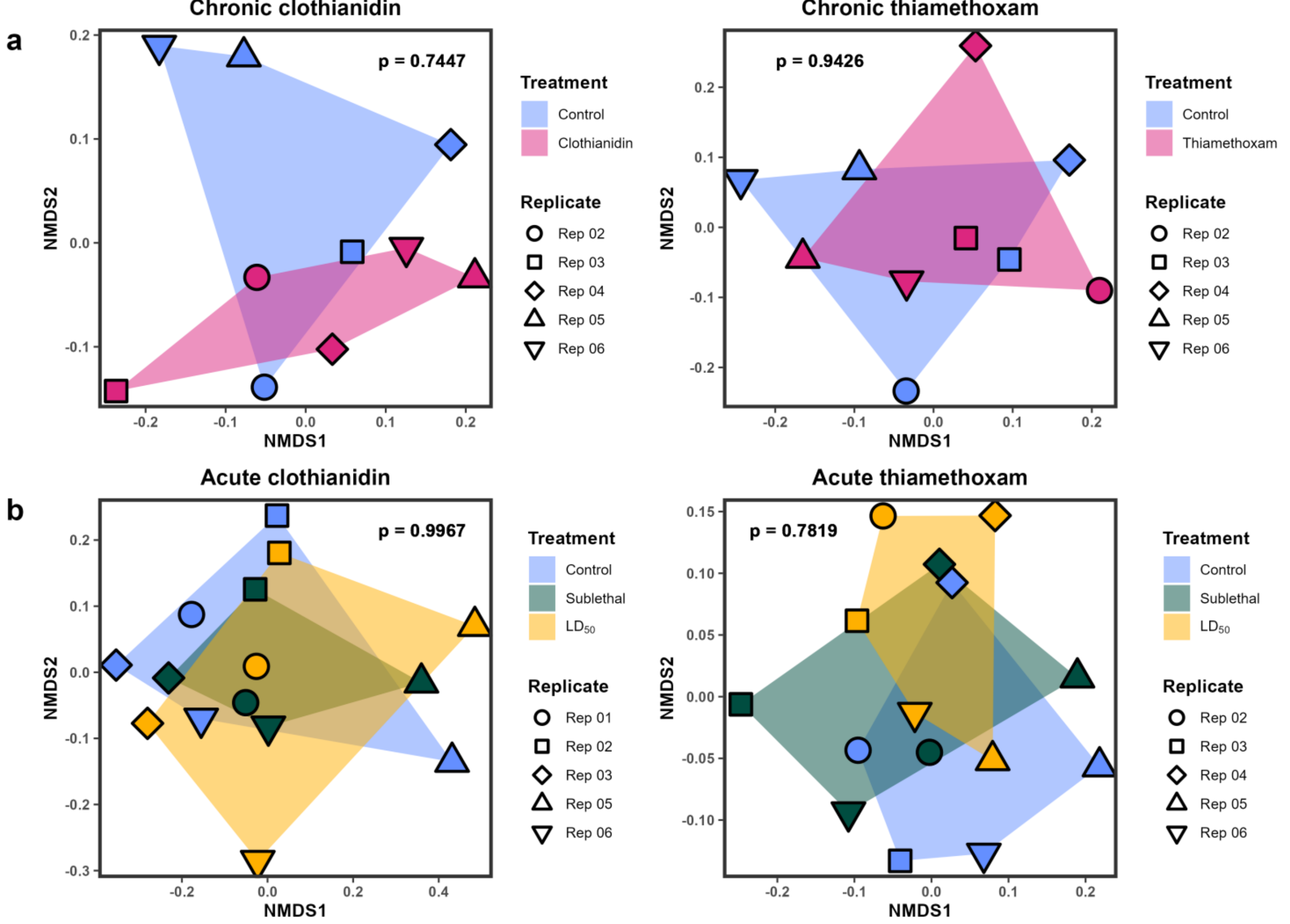

Supplementary Figure S2. NMDS plots using Bray-Curtis dissimilarity to visualize species level beta diversity of honey bees exposed to (a) chronic clothianidin or thiamethoxam in colonies, or (b) exposed to acute clothianidin or thiamethoxam in laboratory cage conditions. Each plot is coloured based on treatment: control (blue), chronic clothianidin/thiamethoxam (red), acute sublethal (red), and acute lethal LD_{50} (yellow). The shapes indicate individual replicates. Dissimilarity was calculated using proportioned raw reads and significance was calculated using ANOSIM with 9,999 permutations. Indicated *p*-values for each plot show the significance of separation grouped by treatment. A *p*-value of less than 0.05 was considered statistically significant.

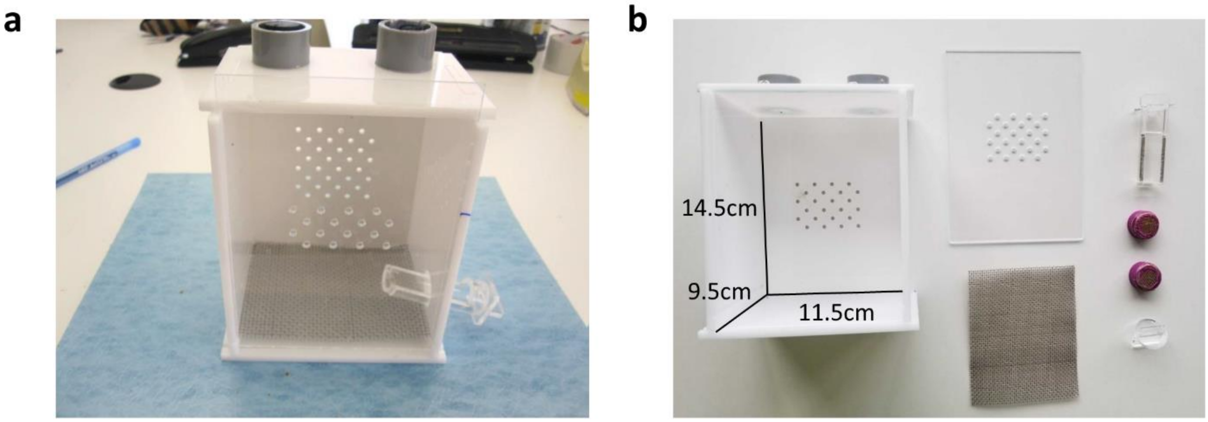

Supplementary Figure S3. Custom cages used for acute sublethal and lethal exposure experiments. (a) An assembled cage. Bees were fed through a conical tube inserted into one of the grey ports at the top with lids modified to accommodate wire mesh though which syrup was fed. (b) Cage components with cage dimensions overlaid. From left to right, top to bottom: cage box, acrylic slider, wire mesh, pollen tray, feeder tube caps, pollen tray lid.
